# MicroRNAs in Esophageal Cancer: Implications for Diagnosis, Progression, Prognosis and Chemoresistance

**DOI:** 10.3390/ijms27020878

**Published:** 2026-01-15

**Authors:** Erica Cataldi-Stagetti, Giulia Governatori, Arianna Orsini, Bianca De Nicolo, Rocco Maurizio Zagari, Elena Bonora

**Affiliations:** 1 Department of Medical and Surgical Sciences (DIMEC), University of Bologna, 40126 Bologna, Italy; erica.cataldi2@unibo.it (E.C.-S.); giulia.governatori6@unibo.it (G.G.); arianna.orsini8@unibo.it (A.O.); roccomaurizio.zagari@unibo.it (R.M.Z.); 2 SSD Medicina e Biologia Molecolare, IRCCS Azienda Ospedaliero-Universitaria di Bologna, 40126 Bologna, Italy; bianca.denicolo2@unibo.it; 3 Gastro-Esophageal Organic Diseases Unit, IRCCS Azienda Ospedaliero-Universitaria di Bologna, 40138 Bologna, Italy

**Keywords:** miRNA, esophageal adenocarcinoma, esophageal squamous cell carcinoma, biomarker, progression, diagnosis, prognosis, chemoresistance

## Abstract

Esophageal cancer (EC), including esophageal squamous cell carcinoma (ESCC) and esophageal adenocarcinoma (EAC), remains a highly lethal disease because of its late diagnosis, significant biological heterogeneity, and frequent resistance to therapy. Growing evidence indicates that microRNAs (miRNAs) are key posttranscriptional regulators involved in tumor initiation, progression, metastasis, and response to treatment. This review provides a comprehensive and updated overview of miRNA dysregulation in both ESCC and EAC, with a specific focus on its emerging clinical relevance in early detection, prognostic assessment, and prediction of therapeutic response. Multiple tissue-based and circulating miRNA signatures, some capable of distinguishing between Barrett’s esophagus (BE), dysplasia, and EAC, demonstrate promising diagnostic performance. In parallel, several miRNAs, including miR-21, miR-23a, miR-455-3p, and miR-196b, have been consistently associated with chemoresistance and radioresistance. Moreover, distinct miRNA expression patterns are correlated with tumor aggressiveness, metastatic potential, and the risk of recurrence, supporting their integration with conventional histopathological and molecular parameters for improved patient stratification. Overall, miRNAs represent a powerful class of biomarkers and potential therapeutic targets in EC, with increasing translational relevance in precision oncology.

## 1. Introduction

Esophageal cancer (EC) is among the most common malignant tumors worldwide and is characterized by high incidence and mortality rates. According to the 2022 Global Cancer Observatory (GLOBOCAN) report, EC ranks as the 11th most frequently diagnosed cancer and the seventh leading cause of cancer-related death globally, accounting for approximately 510,716 new cases and 445,129 deaths in 2022 [1]. These numbers are projected to rise substantially, potentially reaching nearly one million new cases by 2050 [1].

EC comprises two major histological subtypes, esophageal squamous cell carcinoma (ESCC) and esophageal adenocarcinoma (EAC), which display distinct epidemiological, anatomical, and molecular characteristics [2]. ESCC remains the predominant subtype in East Asia, South Central Asia, and southern Africa, whereas EAC is more prevalent in northern Europe and North America [3]. A small minority of esophageal tumors (1–2%) represent other rare histological types, including sarcomas, small-cell carcinomas, melanomas, leiomyosarcomas, carcinoids, and lymphomas [4].

ESCC develops from dysplastic transformation of the squamous epithelium, most frequently in the middle and upper portions of the esophagus. This progression is driven by the accumulation of genetic and molecular alterations that disrupt epithelial integrity and promote uncontrolled proliferation [5]. Early stages involve the transition from normal squamous mucosa to intraepithelial neoplasia (IEN), a recognized precancerous lesion characterized by increasing degrees of dysplasia and genomic instability [6]. Defects in DNA repair and cell cycle regulation arise at this stage and persist throughout tumor evolution, allowing damaged cells to evade normal growth control and acquire malignant potential [7,8]. The primary environmental risk factors for ESCC are tobacco smoking and alcohol consumption. Ethanol enhances mucosal susceptibility by increasing epithelial permeability and facilitating the uptake of tobacco-derived carcinogens; it also induces oxidative stress, DNA damage, and impaired detoxification capacity, fostering genomic instability. Chronic exposure to these agents promotes the selection and expansion of oncogenic clones and malignant transformation [9,10]. Additional cofactors, including advanced age, nutritional deficiencies, and genetic predispositions, further modulate individual risk [11].

In contrast, EAC most commonly originates in the distal esophagus and at the gastroesophageal junction (GEJ). It is strongly associated with gastroesophageal reflux disease (GERD), which causes chronic exposure of the esophageal mucosa to gastric acid and bile salts. This sustained injury induces metaplastic conversion of the normal stratified squamous epithelium into a columnar epithelium resembling the gastric or intestinal mucosa, known as Barrett’s esophagus (BE) [12,13]. BE represents a well-recognized precancerous condition that progresses through stages of metaplasia and dysplasia to invasive adenocarcinoma [12]. Although the malignant potential is greatest in dysplastic BE, even patients without dysplasia have an estimated annual risk of 0.1–0.4% of developing adenocarcinoma [14].

Within this complex sequence of molecular and environmental events, microRNAs (miRNAs) have emerged as pivotal posttranscriptional regulators of gene expression. These small noncoding RNAs (approximately 20–22 nucleotides in length) bind to complementary sequences on target mRNAs, leading to transcript degradation or translational inhibition [15].

Through these mechanisms, miRNAs control key cellular processes, such as proliferation, differentiation, apoptosis, and the stress response [16,17]. In cancer, they act as either oncogenic miRNAs (oncomiRs), which promote tumor growth by repressing tumor-suppressor genes, or tumor-suppressor miRNAs, which inhibit malignancy by targeting oncogenic pathways [18]. It is estimated that miRNAs regulate up to one-third of all human genes, and more than half of known miRNA loci are located within genomic regions associated with cancer or chromosomal fragile sites [19].

Dysregulation of miRNA expression has been increasingly recognized as a hallmark of esophageal carcinogenesis, influencing tumor initiation, progression, metastasis, and response to therapy [20]. Owing to their stability, detectability in body fluids, and broad regulatory capacity, miRNAs also represent particularly attractive candidates as biomarkers for early diagnosis, prognosis, and treatment monitoring, as well as potential therapeutic targets.

In this state-of-the-art review, we provide a comprehensive overview of miRNA dysregulation in esophageal cancer, with particular attention given to the distinct patterns observed in ESCC and EAC. We examine the diagnostic and prognostic utility of miRNAs, as well as their emerging translational implications along the continuum of disease development, progression, and therapy.

## 2. Esophageal Squamous Cell Carcinoma (ESCC)

### 2.1. MicroRNAs as Noninvasive Diagnostic Biomarkers in ESCC

Epigenetic mechanisms, which regulate gene expression without altering the underlying DNA sequence, play a central role in cancer development by influencing tumor initiation, progression, and biologically crucial processes such as proliferation, invasion, metastasis, and drug resistance [21]. Among the various epigenetic regulators, miRNAs have emerged as particularly significant because each miRNA can modulate hundreds of target genes and displays strong tissue-specific expression patterns, thereby making miRNAs powerful tools for early detection, diagnosis, and prognosis across cancer types [22].

#### 2.1.1. Multi-miRNA Signatures for Early Detection

The potential of circulating miRNAs as diagnostic biomarkers for ESCC was first demonstrated in 2010, when Zhang et al. identified a seven-miRNA serum signature (miR-10a, miR-22, miR-100, miR-148b, miR-223, miR-133a, and miR-127-3p) that robustly distinguished patients from healthy controls, including those with early-stage disease [23]. miR-22, miR-133a, and miR-223 act mainly as tumor suppressors by inhibiting cell proliferation, migration and invasion through the modulation of epithelial–mesenchymal transition (EMT)-associated programs and targets such as *SOX4* and *ARTN* [24,25,26]. Other miRNAs in the panel, including miR-10a, miR-100, miR-127-3p and miR-148b, regulate angiogenesis, proliferation and cell motility in different tumor types through pathways involving *VEGF-A*, *FGF2-FGFR2*, *HIF-1α*, mTOR and Wnt/β-catenin signaling and epigenetic or cytoskeleton-associated targets [27,28,29,30]. This discovery was followed by the work of Huang et al., who established a five-miRNA diagnostic panel consisting of miR-20b-5p, miR-28-3p, miR-192-5p, miR-223-3p, and miR-296-5p, all of which are upregulated in ESCC serum [31]. Each miRNA in the panel has been implicated in tumor biology through different mechanisms. miR-20b-5p promotes the activation of the PI3K/AKT pathway by suppressing *PTEN* [32,33]. miR-28-3p is consistently elevated in ESCC tissue, although its downstream pathways remain incompletely characterized [34]. In gastric cancer, miR-28-3p directly targets *ARF6*, a small GTPase that promotes tumor progression via Hedgehog signaling and EMT, suppressing proliferation, migration, and metastasis [35]. miR-192-5p facilitates proliferation by repressing the proapoptotic factor *BIM* [36]; miR-223-3p enhances tumor growth by directly targeting *FBXW7*, a tumor suppressor that controls the stability of key cell cycle regulators such as c-MYC and c-Jun [37]. Downregulation of *FBXW7* leads to the accumulation of these proteins and the consequent activation of the AKT signaling pathway, promoting tumor cell proliferation, survival, and progression [38]. miR-296-5p, initially associated with angiogenesis [39], has shown context-dependent roles, including the suppression of migration and invasion in ESCC [40] and colorectal cancer [41].

Further refinement of miRNA-based diagnostic tools was achieved by Zhou et al., who identified a six-miRNA signature composed of miR-106a, miR-18a, miR-20b, miR-486-5p, and miR-584 (upregulated), along with miR-223-3p (downregulated). This panel demonstrated strong diagnostic accuracy, including for stage I ESCC [42]. The most extensive effort in this field is the work of Sudo et al., who analyzed a large cohort of 566 ESCC patients and 4965 controls to develop the “EC index,” a composite diagnostic score based on six circulating miRNAs (miR-8073, miR-3196, miR-744-5p, miR-6820-5p, miR-6794-5p, and miR-6799-5p) [43]. These miRNAs target genes involved in cell cycle regulation, apoptosis, angiogenesis, EMT, and metastasis and act through pathways such as the TGF-β signaling, PI3K/AKT, and chromatin remodeling pathways [44,45,46,47]. This model achieved outstanding performance, with sensitivity and specificity values of 0.96 and 0.98, respectively, and demonstrated excellent ability to detect stage 0–I tumors. These findings position the EC index as a very promising noninvasive early detection tool [43].

#### 2.1.2. Circulating OncomiRs

Several circulating miRNAs act as oncogenic drivers in ESCC and serve as effective biomarkers because of their consistent upregulation in patient serum. Among them, miR-21 represents one of the most robust and widely validated oncomiRs. Multiple studies [48,49,50,51,52] have shown that miR-21 levels are markedly elevated in ESCC patients and are strongly correlated with tumor size, invasion depth, and aggressive clinical behavior. The levels of miR-21 decrease following surgical resection or chemotherapy and increase again in cases of recurrence, confirming its role as a dynamic and sensitive indicator of tumor burden. Mechanistically, miR-21 promotes oncogenesis by suppressing *PTEN*, *TPM1*, and *PDCD4*, thereby enhancing proliferation and metastatic potential [53].

Another important oncomiR is miR-18a, a member of the miR-17-92 cluster, whose expression is significantly elevated in both tumor tissue and plasma. Its circulating levels decrease postoperatively and increase again during recurrence [54]. Functionally, miR-18a contributes to ESCC development by inhibiting *PTEN* and promoting the expression of cyclin D1, thereby accelerating G1/S cell cycle progression [55].

miR-205-5p, which is detectable in exosomes, also has clinical relevance, as it is significantly enriched in ESCC plasma [56]. This miRNA acts as an oncomiR by directly targeting key tumor suppressors such as *PTEN* and *SMAD4* and activating oncogenic pathways, including the PI3K/AKT and TGF-β signaling pathways. Through these mechanisms, miR-205 has been widely implicated in promoting tumor initiation and EMT in multiple cancer types [57,58,59,60].

Other circulating miRNAs upregulated in ESCC include miR-25 [61], which supports tumor progression by repressing several tumor-suppressor genes and activating the *TGF-β*, *VEGFA/VEGFR*, *EGF/EGFR*, and Wnt/β-catenin signaling pathways, which are fundamental for angiogenesis and metastasis [62]. Additional oncogenic biomarkers include miR-1322, which regulates the tumor-suppressor gene *ECRG2* in an allele-specific manner [63], and the triad, miR-16-5p, miR-451a, and miR-574-5p, each of which exhibits strong diagnostic performance, with AUC values exceeding 0.80 [64]. Although their biological functions differ, with miR-16-5p promoting tumor growth through *RECK* and *SOX6* suppression [50], miR-451a regulating erythroid maturation [65], and miR-574-5p acting as an oncogene in multiple cancers [66], their collective upregulation provides additional support for their use as circulating biomarkers.

#### 2.1.3. Tumor-Suppressive miRNAs

Several circulating miRNAs function as tumor suppressors and are consistently downregulated in ESCC.

miR-375 was one of the earliest identified miRNAs to be significantly reduced in the plasma of ESCC patients [48]. Its known involvement in proliferation, invasion, metastasis, and therapeutic resistance [67,68] suggests that its dysregulation may mark the onset of malignant transformation.

Similarly, miR-216a and miR-216b are significantly downregulated in patient serum [69] and are known to inhibit oncogenic pathways across multiple tumor types by targeting *KRAS*, *IGF2BP2*, *eIF4B*, and *ZEB1* [70,71,72]. The functional role of miR-216a appears context dependent, as it has been shown to act as an oncogene in early hepatocellular carcinoma [73], emphasizing the need for cancer-specific mechanistic studies in ESCC.

A particularly relevant tumor-suppressive miRNA for ESCC is miR-613, which was previously described in thyroid and gastric cancer [74,75]. miR-613 is significantly downregulated in ESCC tissues and patient serum [76]. Mechanistically, miR-613 has been shown to inhibit tumor cell invasion and migration by directly targeting fibronectin 1 (*FN1*), a key extracellular matrix component involved in cell adhesion, motility, and metastatic dissemination [77]. In ESCC, miR-613 downregulation can be further exacerbated by CircRIMS, a circular RNA markedly overexpressed in this cancer, which suppresses miR-613 expression through a DNMT1-dependent DNA methylation mechanism, thereby promoting tumor cell proliferation both in vitro and in vivo [78].

Another emerging tumor suppressor is miR-1297, which was first investigated in the serum of patients with ESCC by Wang et al. The study revealed significant downregulation of miR-1297 in patient serum compared with healthy controls, with excellent diagnostic accuracy. Importantly, even early-stage patients presented AUC values above 0.81, with sensitivity and specificity surpassing 80% [79]. Mechanistically, miR-1297 directly targets *EphA2*, a receptor tyrosine kinase associated with metastasis and poor clinical outcomes [80]. Recent evidence has demonstrated that the early overexpression of *EphA2* in premalignant breast lesions results from the loss of miR-1297, suggesting that a conserved tumorigenic axis that may also be relevant in ESCC [81].

### 2.2. MicroRNAs Involved in the Development and Progression of ESCC

miRNAs play crucial regulatory roles in ESCC progression through their ability to fine-tune posttranscriptional gene expression programs that control invasion, metastasis, EMT, proliferation, and cell survival. Their biological importance is particularly evident in ESCC, a malignancy typically diagnosed at advanced stages and characterized by aggressive invasive behavior [82]. Importantly, circulating miRNAs demonstrate exceptional stability in serum and plasma and exhibit consistent, reproducible expression across individuals, underscoring their value as noninvasive biomarkers suitable for early diagnosis and real-time disease monitoring [83,84].

In this section, we examine the contribution of dysregulated miRNAs to ESCC progression, focusing on those that are downregulated and function as tumor suppressors, as well as those that are upregulated and act as oncogenic drivers promoting aggressive tumor behavior and metastatic dissemination.

#### 2.2.1. Tumor-Suppressive miRNAs in ESCC Progression

A substantial subset of miRNAs is downregulated in ESCC, leading to key malignant traits. Among the most relevant miRNAs, miR-29b is markedly reduced in ESCC tumor tissues and cell lines; its reintroduction suppresses in vitro invasiveness and slows xenograft growth in vivo by directly inhibiting *MMP-2*, a matrix metalloproteinase essential for ECM degradation and metastatic spread [85]. Consistent with observations in osteosarcoma, uterine leiomyoma and ovarian carcinoma, reduced miR-29b expression in ESCC is associated with increased invasive potential, particularly lymph node metastasis [86,87,88].

Another crucial tumor suppressor is miR-30c, which is significantly downregulated in both tumor tissues and the serum of ESCC patients [89,90]. Similar reductions have been described in other malignancies [91,92,93], further supporting its tumor-suppressive function. Restoration of miR-30c in ESCC cells reduces proliferation and migration while increasing E-cadherin and decreasing N-cadherin and vimentin, blocking EMT through the repression of *SNAI1*. Recent data also support its ability to modulate the PI3K/AKT pathway [89,90].

miR-150 is another regulator of the EMT/MET (mesenchymal–epithelial transition) axis. Its expression is significantly reduced in ESCC, and its re-expression induces a MET-like phenotype, suppressing migration, invasion, and tumorigenicity in vivo. Functionally, miR-150 targets *ZEB1*, a key EMT driver implicated in stem-like tumor cell features [94]. Several of its known targets, including *c-MYB*, *NOTCH3*, and *CXCR4*, have long been recognized as key regulators of cancer progression, reinforcing the relevance of miR-150 loss in ESCC aggressiveness [95,96,97].

miR-375 plasma levels are significantly lower in patients than in healthy controls [48,52]. Its tumor-suppressive role is well established across several malignancies, including head and neck squamous cell carcinoma [98], hepatocellular carcinoma [99], and gastric cancer [100], where it regulates multiple oncogenic pathways [101]. Functional studies have demonstrated that miR-375 suppresses proliferation, colony formation, and metastasis both in vitro and in vivo [56] by directly inhibiting multiple oncogenic genes, including *PRDX1* [102], *IGF1R* [103], and *XPR1* [104]. PRDX1, a redox-modulating protein, promotes tumor cell growth and survival by modulating reactive oxygen species and supporting oncogenic signaling [102]. *IGF1R*, encoding a key regulator of the PI3K/AKT pathway, is negatively regulated by miR-375, leading to reduced proliferation, survival, and metastasis [103]. XPR1, a phosphate exporter and receptor with viral binding activity, can increase proliferation, migration, and invasion through the activation of oncogenic signaling pathways, including the BRAF-ERK1/2-p53 pathway, and the modulation of phosphate homeostasis [105].

Another robust tumor suppressor, miR-486-5p, is strongly downregulated in ESCC [34,106]. It represses critical regulators of cell cycle progression and survival, including *CDK4*, which promotes the G1–S transition via E2F activation, and *BCAS2*, encoding a PRP19-complex component involved in mRNA splicing and p53 regulation whose loss disrupts cell division and promotes apoptosis [107].

Similarly, miR-630 is downregulated in ESCC and acts as a potent inhibitor of proliferation and invasion [108,109]. Functional studies have shown that miR-630 restoration suppresses EMT-associated invasive traits by directly targeting *SLUG*, a master regulator of EMT, leading to the re-expression of epithelial markers such as E-cadherin and β-catenin and a concomitant reduction in mesenchymal markers, including N-cadherin and vimentin [108]. miR-630 exerts tumor-suppressive functions across multiple solid malignancies, including pancreatic, ovarian, lung, and prostate cancers, by inducing apoptosis and limiting tumor aggressiveness through the inhibition of key oncogenic targets [110,111,112,113]. These include GAK (cyclin G-associated kinase), which is involved in clathrin-mediated membrane trafficking [111]; PRKCI, whose repression impairs proliferation, clonogenicity, and cancer stem–like properties via the suppression of Hedgehog signaling [113]; and components of the JAK2/STAT3 pathway, resulting in reduced vimentin expression and invasive capacity [112].

Circulating miR-718 is also decreased in ESCC: its plasma levels are significantly lower in patients than in healthy controls and increase postesophagectomy [114], suggesting a close association with tumor burden and dissemination. Although its molecular targets in ESCC remain undefined, evidence from ovarian cancer indicates that miR-718 directly targets *VEGF*, leading to the inhibition of tumor cell proliferation, invasion, and angiogenesis and increased apoptosis [115]. In addition, miR-718 has been reported to suppress tumor growth in hepatocellular carcinoma by targeting *HOXB8*, which encodes for a transcription factor involved in tumorigenesis and metastasis [114].

#### 2.2.2. Oncogenic miRNAs in ESCC Progression

On the other hand, several miRNAs are upregulated in ESCC and function as strong promoters of malignant progression. Among them, miR-9 is markedly elevated in tumor tissues and patient plasma and is associated with advanced invasive and metastatic phenotypes [116,117]. Functionally, miR-9 induces EMT by suppressing α1-catenin, enhancing β-catenin nuclear translocation, and activating downstream targets such as VEGF, c-MYC and CD44, thereby reducing E-cadherin, increasing the expression of mesenchymal markers, including vimentin and fibronectin, and promoting invasion and metastasis [118,119].

miR-155-5p is another well-established oncomiR that is consistently overexpressed in ESCC [120]. It promotes proliferation and migration while inhibiting apoptosis by targeting *TP53INP1*, impairing p53 function and enabling uncontrolled cell cycle progression [121,122]. This leads to accelerated G1–S transition in ESCC cells, sustaining continuous proliferation [123].

Similarly, miR-183 enhances tumor aggressiveness. Its overexpression increases the proliferation, migration, invasion, and survival of ESCC cells, whereas its inhibition reverses these effects. Mechanistically, miR-183 directly represses *PDCD4*, a proapoptotic gene, contributing to tumor progression [124,125]. Furthermore, c-Jun directly binds the miR-183 promoter and upregulates its expression; increased miR-183 then targets *SMAD4*, promoting EMT through the upregulation of N-cadherin and vimentin and the downregulation of E-cadherin [126].

Circulating miR-367 is increased in advanced ESCC patients and decreases significantly after surgery or chemotherapy [127]. Although its direct role in ESCC remains to be fully clarified, studies in bladder carcinoma have demonstrated that the miR-367-3p/RAB23 regulatory axis drives tumor proliferation, migration, and invasion [128].

A recent comprehensive analysis by Tanaka et al. provided important insights into the miRNA-mediated modulation of ESCC differentiation and aggressiveness. This study highlighted miR-100-5p and miR-203a-3p as key factors involved in the inhibition of ESCC cell migration and invasion [129]. Previous research has shown that miR-100-5p suppresses *CXCR7* and inhibits PI3K/AKT signaling [130,131], whereas miR-203a-3p exerts a similar inhibitory effect on the PI3K/AKT pathway [132]. In this signaling context, Tanaka et al. identified *FKBP5* as a crucial convergent target of both miR-100-5p and miR-203a-3p. FKBP5 acts as an oncogenic mediator in several malignancies by enhancing tumor progression and chemoresistance through NF-κB activation [133]. In ESCC, it also amplifies PI3K/AKT signaling, further promoting aggressive behavior. These findings point to FKBP5 as a promising therapeutic target and suggest that the combined restoration of miR-100-5p and miR-203a-3p may represent an effective strategy to block ESCC progression [129].

### 2.3. MicroRNAs as Prognostic Biomarkers in ESCC

Despite advances in diagnostic and therapeutic strategies, ESCC recurrence rates are high, and long-term survival remains limited. In this context, the identification of molecular biomarkers that are able to provide reliable prognostic information is crucial. Several studies have demonstrated that specific alterations in miRNA expression are closely associated with the clinical outcome of patients with ESCC.

One of the most extensively investigated miRNAs in ESCC as a negative prognostic biomarker is miR-21. In several studies, miR-21 was significantly upregulated in tumor tissues compared with adjacent nonneoplastic tissues, particularly in stage II carcinomas. High miR-21 expression is associated with unfavorable clinicopathological features, including poorer tumor differentiation, lymph node metastases, and reduced progression-free and overall survival in patients undergoing surgical resection [134,135]. miR-21 exerts its oncogenic effects by repressing the tumor suppressor gene *PDCD4*, whose low expression in tumor tissues is in turn associated with worse clinical outcomes [135,136].

In contrast, miR-133a acts as a tumor suppressor and a favorable prognostic marker in ESCC. Its expression is significantly downregulated in ESCC tissues compared with adjacent normal tissues, and low miR-133a levels are associated with more aggressive clinicopathological features, including advanced tumor stage and increased tumor length, as well as significantly reduced overall survival and disease-free survival [26,137]. miR-133a inhibits cell proliferation, migration and invasion by regulating genes involved in EMT, such as *SOX4*, and receptors promoting tumor growth, including *IGF1R* [138].

Similarly, miR-133b is considered a strong tumor suppressor in ESCC, with reduced expression associated with a poorer prognosis and a more aggressive tumor phenotype [139]. Low miR-133b levels correlate with increased phosphorylation of STAT3 (p-STAT3) and deregulation of the JAK2/STAT3/Bcl-2 axis, thereby promoting cell survival and tumor progression [140]. In addition, the lncRNA TTN-AS1 acts as a molecular “sponge” for miR-133b, reducing its functional availability and consequently increasing the expression of *SNAIL1*, a key regulator of EMT, thus further promoting invasion and metastasis [141].

Another miRNA with high prognostic value in ESCC is miR-138, which is significantly downregulated at both the tissue and serum levels in affected patients. Its reduced expression is associated with adverse clinicopathological parameters, lymph node metastasis and increased tumor aggressiveness, thus representing a negative prognostic marker [142,143]. Similar findings have been reported in other solid tumors, including thyroid cancer [144], non-small cell lung cancer [145] and osteosarcoma [146], in which loss of miR-138 was associated with metastasis and poorer survival. It has been proposed that miR-138 loss promotes lipid raft formation and constitutive activation of the NF-κB pathway, resulting in enhanced proliferation, invasion and survival of tumor cells [142].

miR-375 has also been extensively described as a prognostic biomarker in ESCC [103,147,148]. Because it is known for its tumor-suppressive function by inhibiting ESCC cell proliferation, colony formation and metastasis both in vitro and in vivo [56], miR-375 has also been identified as an independent prognostic factor. In particular, a meta-analysis by Luo and Wu demonstrated that low miR-375 expression is associated with reduced overall survival. This effect may be mediated, at least in part, by the downregulation of PDK1, a key kinase in the PI3K/AKT signaling pathway, resulting in the inhibition of PI3K/AKT signaling, reduced aerobic glycolysis and decreased tumor growth [148,149].

miR-1246 has emerged as one of the most clinically informative oncomiRs and one of the strongest independent predictors of poor survival in patients with ESCC. Elevated serum levels of miR-1246 are associated with advanced tumor stage (T3–T4), lymph node and distant metastases, and stage III–IV disease. Since its expression decreases postoperatively, it has been proposed for posttreatment surveillance. MiR-1246 modulates the p53 network, inhibits *DYRK1A* and modulates stem-like characteristics associated with invasion and adhesion [150].

miR-655, which is implicated in EMT regulation and ESCC prognosis [151], is markedly downregulated in ESCC cells and tissues, and its overexpression increases E-cadherin levels by directly targeting *ZEB1* and *TGFBR2*, thereby significantly inhibiting cell migration and invasion [152]. Moreover, miR-655 is downregulated at the plasma level, particularly in exosomes, and reduced circulating levels are associated with lymph node metastasis, lymphatic invasion and advanced pathological stage. Notably, this association is already present in early-stage disease, suggesting a potential role for miR-655 as an early prognostic predictor and a risk stratification marker [153].

In addition, several other miRNAs have been identified as potential prognostic biomarkers in ESCC. miR-203 represents a favorable prognostic factor: its high expression is associated with improved survival and reduced tumor growth, promoting squamous differentiation and repressing key proliferation regulators such as p63 [154]. In contrast, miR-129 and miR-296 are associated with an unfavorable prognosis. High levels of miR-129 correlate with reduced postoperative survival and may contribute to tumor progression by targeting tumor suppressors such as *APC* and *RAB11* [155]. Similarly, high miR-296 expression is associated with worse outcomes and increased tumor aggressiveness and chemoresistance through the modulation of cyclin D1, p27, and the drug efflux glycoprotein MDR1 (P-gp) [156]. Finally, miR-142-3p has been identified as an independent negative prognostic factor, even in patients with early-stage disease; its high expression correlates with poor cellular differentiation and with the regulation of genes involved in proliferation, adhesion and apoptosis, including *APC*, *KLF4* and *BCL2L1* [157].

### 2.4. MicroRNAs as Biomarkers for Cancer Resistance in ESCC

Drug resistance is a major cause of tumor recurrence. In the case of ESCC, the clinical manifestations in the early stages are modest and nonspecific; therefore, many patients are diagnosed in intermediate or advanced stages of the disease, effectively compromising their suitability for optimal surgical treatment [158]. Radiotherapy and chemotherapy, particularly 5-fluorouracil (5-FU), cisplatin (CDDP), and taxanes, remain standard therapeutic approaches [159]; however, treatment efficacy and overall survival remain limited, highlighting the need to clarify the mechanisms of the therapeutic response and identify reliable predictive biomarkers. In this context, circulating miRNAs represent a particularly promising biomarker class. The use of these minimally invasive methods, combined with increasing evidence that they regulate tumor cell sensitivity to antineoplastic agents, underscores their potential as predictors of therapeutic response.

miR-27a is one of the earliest examples of drug resistance, since its downregulation enhances the sensitivity of ESCC cell lines to both P-gp and non–P-gp substrate drugs, potentiates ADR-induced apoptosis, and reduces P-gp, BCL2, and MDR1 levels while increasing BAX, shifting the BCL2/BAX ratio toward apoptosis [160].

miR-148a plays a chemosensitizing role: its upregulation after transfection increases ESCC sensitivity to 5-FU and cisplatin and attenuates resistance in chemotherapy-resistant cell lines. This effect is mediated through the modulation of multiple pathways conferring chemoresistance, including epigenetic regulation via DNA methyltransferases (DNMT1 and DNMT3B), stress–response signaling through MSK1, and drug metabolism and efflux by targeting PXR, a key regulator of ABC transporters [161].

In contrast, miR-141 promotes cisplatin resistance by targeting the 3′-UTR of *YAP1*, a key mediator of genotoxic agent-induced apoptosis, thus resulting in downregulation of its expression [162].

High plasma levels of miR-23a represent an independent risk factor for chemoresistance in ESCC. Through a genome-wide analysis of plasma miRNAs from ESCC patients, increased miR-23a expression was found in the pretreatment plasma and ESCC tissues of patients with a limited response to therapy. The overexpression of miR-23a also induced marked resistance to 5-FU and cisplatin in vitro. Although its downstream mechanisms in ESCC are not fully defined, miR-23a has been shown to target multiple tumor suppressor genes, including *PTEN*, *CDH1*, *IRF1*, *APAF1*, and *TOP2B*, thereby promoting prosurvival signaling, EMT-related programs, and resistance to cytotoxic treatments [163].

In contrast, miR-218 is downregulated in cisplatin-resistant ESCC cells, and its overexpression restores sensitivity to treatment. miR-218 indeed increases cisplatin sensitivity in esophageal tumor cells by modulating survivin expression [164].

The expression levels of miR-455-3p in tumor tissues significantly correlated with patient overall survival (OS) and recurrence-free survival (RFS). The overexpression of miR-455-3p led to a marked increase in the subpopulations of CD90+ and CD271+ tumor-initiating cells (T-ICs), which were previously identified as T-ICs in ESCC [165,166]. Notably, treatment with a specific antagomir against miR-455-3p not only increased the sensitivity of ESCC cells to chemotherapy but also reduced the number of T-IC subpopulations. Furthermore, aberrant expression of miR-455-3p in ESCC cells has been reported to activate the Wnt/β-catenin and TGF-β/SMAD signaling pathways through the concurrent suppression of multiple negative regulators of these pathways [167].

miR-21 is highly expressed in patients with ESCC and is also closely associated with tumor stage, lymph node metastasis, and the inflammatory response [168]. Several studies have suggested that miR-21 could be used as a biomarker to predict patient resistance to chemotherapy [169]. In particular, Wang et al. reported that ESCC patients with high miR-21 expression (>5.80) have a significantly increased risk of ineffective treatment, suggesting the potential predictive role of miR-21 in the therapeutic response [170].

miR-29c represents another potential predictive biomarker. Its reduced expression in the tumors and serum of ESCC patients is correlated with chemoresistance, while restoration of miR-29c resensitizes chemoresistant ESCC cells (KYSE150FR and KYSE410FR) to 5-FU. miR-29c directly targets *FBXO31*, thereby modulating stress–response signaling pathways, including the p38 MAPK axis, which are implicated in cell survival and drug resistance [171].

miR-196b promotes broad chemoresistance (to UV, cisplatin, paclitaxel, 5-FU, and epirubicin) through *EPHA7* inhibition and *EPHA2*-driven EMT activation [172].

In the case of miR-192-5p, elevated levels correlate with chemosensitivity. Plasma miR-192-5p levels can predict the response to neoadjuvant chemotherapy and the prognosis of patients with esophageal cancer [173]. In particular, Furuke et al. reported that its downregulation in KYSE170 cells reduces cisplatin sensitivity, whereas its overexpression in the human cell line TE-15 enhances sensitivity via the modulation of *ERCC3* and *ERCC4*, key genes involved in DNA repair–mediated resistance [174].

**Figure 1:**
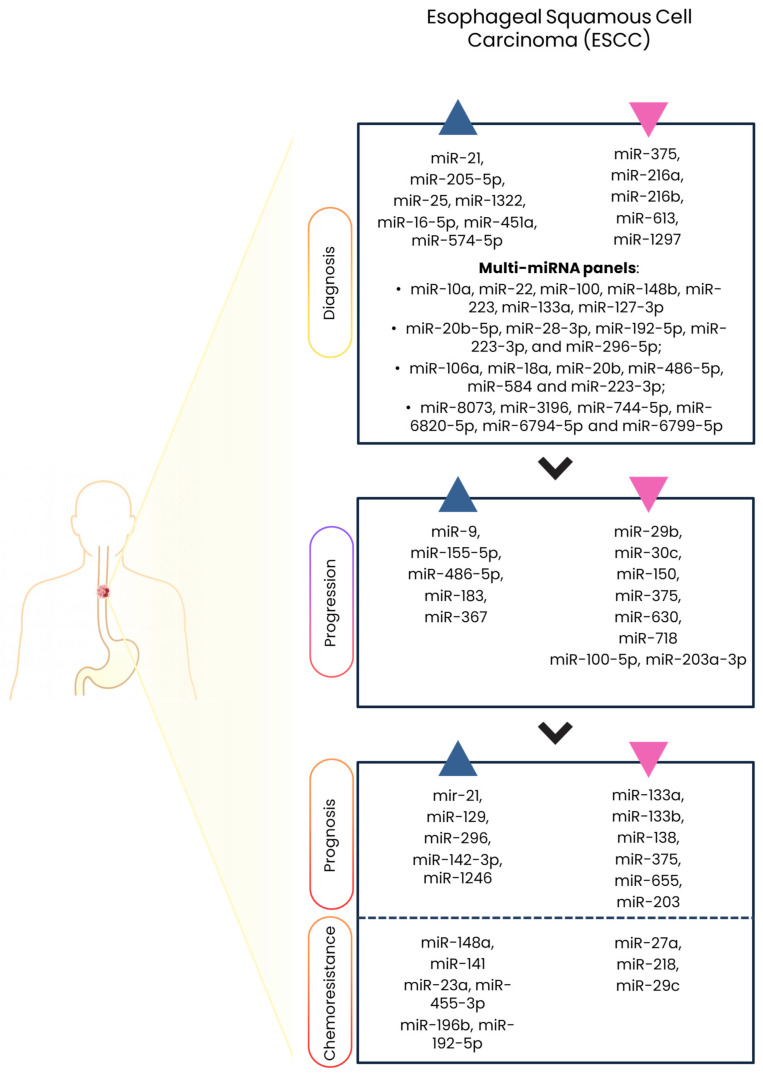
Summary of miRNA dysregulation in ESCC. miRNAs that are upregulated are indicated with blue arrowheads, and miRNAs that are downregulated are indicated by pink arrowheads; these miRNAs are associated with early detection and diagnosis, progression, prognosis, and resistance or sensitivity to chemoradiotherapy in ESCC. (Created in BioRender. Cataldi-Stagetti, E. (2026) https://BioRender.com/o090zbo, accessed on 17 December 2025).

A summary of the dysregulated miRNAs implicated in ESCC diagnosis, progression, prognosis, and therapy resistance is provided in Table 1 and Figure 1.

## 3. Esophageal Adenocarcinoma (EAC)

### 3.1. MicroRNAs as Noninvasive Diagnostic Biomarkers in EAC

Despite intensive screening with current techniques, over 80% of EACs are diagnosed in patients without a prior diagnosis of BE or GERD [175,176]. Moreover, more than 80% of BE cases in the general population remain undiagnosed, thereby failing to benefit from surveillance strategies [177]. These limitations highlight the urgent need for molecular biomarkers able to identify individuals at high risk of progression and distinguish true progressors from nonprogressors.

High-throughput profiling studies have shown that miRNA expression patterns can reliably distinguish BE, dysplasia, and EAC, supporting their potential as promising tools for molecular diagnosis.

Among the miRNAs altered in BE, miR-194 shows a particularly consistent pattern: its HNF-1α–mediated upregulation aligns with the acquisition of the intestinal phenotype [178]. Several studies have reported a progressive increase in miR-194 from columnar metaplasia to EAC, making it a sensitive indicator of early BE development [178,179]. Wijnhoven, Cabibi and colleagues demonstrated that the levels of miR-194 and miR-215, together with those of miR-143 and miR-145, increase progressively across the stages that precede BE, i.e., esophagitis and columnar metaplasia, and reach their highest levels in established BE, both in tissue and in serum. These miRNAs are linked to regulatory networks controlling epithelial differentiation, cell cycle regulation (via p53), and responses to genomic stress and represent reliable markers of the initial transition toward BE metaplasia before dysplastic changes arise [180,181].

In contrast, miRNAs typical of squamous differentiation exhibit the opposite behavior. miR-203 and miR-205 are downregulated in BE and further reduced in EAC, defining an inverse gradient that reflects the progressive loss of squamous features. In particular, miR-203 downregulation reactivates *TP63* expression, facilitating the metaplastic switch toward a columnar phenotype [180].

Circulating miRNAs also offer diagnostic potential. Bus et al. reported that the levels of miR-194-5p, miR-451a and miR-365a-3p are elevated in the plasma and tissue of BE/EAC patients, supporting their utility as noninvasive biomarkers [182]. Moreover, Slaby et al. identified a two-miRNA tissue signature (miR-375 and miR-93) capable of differentiating nondysplastic BE (BE-ND), dysplastic BE, and EAC with high accuracy (AUC 0.89), providing objective support for routine FFPE histological assessment [183].

A large-scale analysis further expanded this diagnostic landscape, identifying 46 miRNAs significantly upregulated in EAC compared with BE. A subset, miR-663b, miR-421, and miR-502-5p, was detected in more than 80% of EACs but in fewer than 20% of BE biopsies, forming a highly discriminative signature. Several of these miRNAs target well-known tumor suppressors or oncogenes, including *SMAD4*, *PTEN*, *c-MYC* and *BCL2*, reinforcing their diagnostic relevance [184].

Serum-based profiling by Zhang et al. revealed a dysregulated circulating miRNA pattern in EAC, with miR-25 and miR-151 significantly upregulated and miR-100 and miR-375 markedly reduced. These four miRNAs influence key oncogenic and tumor-suppressive pathways, including the CDH1, p21, Bim, PDK1, IGF1R, MXI1 and JAK2 pathways, which regulate cell cycle progression, apoptosis, proliferation, metabolic adaptation, clonogenicity, invasion, and metastatic potential, making them promising panels for EAC screening [185].

Recently, the EMERALD study developed and validated a six-miRNA plasma signature for the noninvasive detection of BE (with or without dysplasia) and EAC. The expression levels of miR-106b, miR-146a, miR-15a, miR-18a, miR-21 and miR-93 reliably distinguished BE, low-grade dysplasia (LGD), high-grade dysplasia (HGD) and EAC from both healthy controls and GERD patients. Within this panel, the most robust contributors, miR-21-5p, miR-93-5p and miR-15a-5p, participate in the regulation of cell cycle progression and apoptotic sensitivity [186].

These findings indicate that circulating miRNAs could become powerful, minimally invasive tools to aid early detection and risk stratification in patients with BE and EAC.

### 3.2. MicroRNAs Involved in the Development and Progression of EAC

Several miRNAs exhibit expression patterns and biological functions consistent with roles in the progression from BE to dysplasia and invasive EAC, contributing to EMT, invasion, apoptosis resistance and metastasis.

Among them, miR-196a is one of the earliest and most robust markers of progression. Maru et al. demonstrated a dramatic increase in miR-196a levels in early BE, with a further gradual increase through LGD, HGD and EAC, reaching levels 10–100-fold greater than those in nonneoplastic tissue. This pattern indicates that miR-196a deregulation is both an early and persistent carcinogenic event and that its marked upregulation in HGD may help distinguish LGD from HGD [183,187]. Functionally, miR-196a promotes malignant transformation by repressing genes typically lost in EAC (*SPRR2C*, *S100A9* and *KRT5*) and enhances proliferation and EMT through the modulation of the NF-κB and c-MYC pathways [187,188].

Leidner and collaborators examined 26 miRNAs across the BE–dysplasia–EAC sequence, identifying two, miR-31 and miR-375, as true progression markers. The expression of miR-31, which was largely unchanged in BE-ND, was sharply downregulated in HGD and EAC. Early loss of miR-31 in BE correlates with an increased risk of neoplastic progression, which is consistent with a tumor-suppressive role involving the E2F2/CDKN2A axis. In contrast, miR-375 is selectively lost only in EAC, not in BE or dysplasia, marking the transition to invasive carcinoma and affecting targets such as PDK1 and IGF1R, which promote metabolic adaptation and proliferation [189].

A subsequent analysis revealed a five-miRNA signature marking the key steps of the progression sequence: miR-215, miR-192, miR-205, let-7c and miR-203. p53-induced miR-192 and miR-215 are progressively upregulated from metaplasia through dysplasia to carcinoma, whereas miR-205, miR-203 and let-7c undergo gradual downregulation. Loss of miR-203 and miR-205 reflects the gradual loss of differentiation of BE; downregulation of let-7c, which normally represses *HMGA2*, contributes to the increased expression of this oncogenic protein during progression [190].

miR-21, one of the most frequently upregulated oncomiRs in cancer, also functions as a progression marker in BE–EAC [191]. Its expression increases from BE to carcinoma, and functional studies in EAC have shown that miR-21 enhances anoikis resistance, which is crucial for metastatic spread, by directly suppressing *PDCD4* and *PTEN*, thereby activating PI3K/AKT signaling [136].

Finally, an important contribution to early progression comes from the circulating miRNAs identified by Fassan and colleagues. In the serum of HGD patients compared with that of BE-ND patients, seven miRNAs were upregulated (miR-92a-3p, miR-151a-5p, miR-362-3p, miR-345-3p, miR-619-3p, miR-1260b, and miR-1276), and three were downregulated (miR-381-3p, miR-502-3p, and miR-3615). Among these, miR-92a-3p has emerged as the most significant marker of early progression. In both the serum and tissue of neoplastic patients, miR-92a-3p belongs to the oncogenic miR-17-92 cluster and modulates key targets such as *PTEN*, *BIM* and components of the TGF-β/SMAD pathway [192].

### 3.3. MicroRNAs as Prognostic Biomarkers in EAC

EAC is a highly aggressive and biologically heterogeneous malignancy characterized by marked genomic instability and an overall poor prognosis. This heterogeneity hampers the identification of reliable prognostic markers and limits the implementation of truly personalized therapeutic strategies.

Recently, the use of advanced high-throughput sorting techniques combined with massive sequencing has been shown to enable a more accurate characterization of the mutational landscape and tumor heterogeneity than the analysis of bulk tissues [193]. In addition, a diagnostic algorithm based on the glandular architectural features of EAC has been developed, allowing the distinction and subclassification of specific histological subtypes; when combined with pathological stage, this algorithm has high prognostic power, enabling a more accurate discrimination of patients at low or high risk of cancer-specific mortality [194]. In this framework, the potential contribution of miRNAs as complementary prognostic markers was also assessed [195]. In that study, two miRNAs, miR-221 and miR-483-3p, which are known to promote growth and progression in several solid tumors [196,197,198,199,200], were identified as being consistently overexpressed in EAC samples. In particular, miR-221 overexpression was significantly associated with worse cancer-specific survival and an increased risk of recurrence, with a clinically evident impact even in EAC subgroups classified as low risk according to the EACSGE classification [194]. In parallel, the lower expression of miR-483-3p in early-stage disease suggests its potential utility in the initial stratification of EAC. At the molecular level, the deregulation of these miRNAs is associated not only with the modulation of key genes involved in invasiveness, metabolism, and drug response but also with altered expression of the oncogenic lncRNA *MALAT1*, a well-known regulator of metastasis, cell survival, and apoptosis [195].

A contribution to prognostic stratification in EAC was provided by Matsui et al. In a cohort of stage I patients, four miRNAs, namely, miR-652-5p, miR-7-2-3p, miR-3925-3p, and miR-219-3p, were identified as significantly associated with disease progression. The clinical relevance of this signature was subsequently validated in an independent cohort of stage II/III patients, in which miR-652-5p and miR-7-2-3p emerged as independent prognostic markers. In particular, reduced expression of these two miRNAs was associated with poorer overall survival and progression-free survival, suggesting their potential use in identifying high-risk patients who may benefit from adjuvant therapy [201]. miR-652-5p downregulation promotes tumor aggressiveness via hypermethylation of its promoter and activation of the SDC1/TGFβ2/pERBB4 axis under hypoxic conditions [202]; miR-7-2-3p acts as a tumor suppressor by targeting *DCLK1*, thereby modulating the PI3K/Akt/NF-κB pathway and limiting proliferation, EMT, and metastatic potential [203].

Several other miRNAs with prognostic significance in EAC have been identified. Gu et al. conducted one of the largest studies on circulating miRNAs in EAC, analyzing a cohort of 72 patients in the discovery phase and 329 patients in the validation phase. In both cohorts, a significant association emerged between high serum levels of miR-331-3p and a markedly reduced risk of recurrence (−55% and −45%, respectively). This study is the first to report the ability of miR-331-3p to stratify recurrence risk accurately in patients with EAC [204]. The expression of miR-331-3p, a known tumor suppressor, is also downregulated in other solid malignancies, including gastric [205], prostate [206], thyroid [207], and breast cancer [208]. Its overexpression is associated with the inhibition of cell proliferation and migration, and with reduced activity of the HER2 receptor, a direct target of this miRNA, leading to the inhibition of the PI3K/AKT pathway. Since *HER2* gene is frequently amplified in EAC and contributes to tumor aggressiveness and metastatic dissemination, its downregulation represents a plausible mechanism underlying the protective effect of miR-331-3p against recurrence [204].

In addition to their role in recurrence and disease progression, several circulating miRNAs also appear to be directly associated with mortality risk in patients with EAC. In 2021, Petrick and colleagues identified 79 circulating miRNAs that are differentially expressed between EAC patients and healthy controls. The most relevant finding was the identification of two miRNAs, miR-4253 and miR-1238-5p, that are significantly associated with postdiagnosis mortality in EAC patients. These miRNAs influence key oncogenic and tumor-suppressive pathways involved in cell proliferation, apoptosis, migration and invasion by acting through targets such as *LHX2* and other effectors of oxidative stress, growth arrest, and survival signaling [209]. miR-4253 has previously been linked to aggressiveness in HER2-positive breast cancer [210] and to mechanisms related to oxidative stress and apoptosis [211]. miR-1238-5p expression has been associated with proliferation and invasion in osteosarcoma [212] and with chemoresistance in glioblastoma [213].

Odenthal and colleagues analyzed the miRNA profile in the sera of patients with EAC undergoing multimodal treatment followed by surgical resection. This study highlighted a significant prognostic role for miR-222 and miR-302c. Specifically, high expression of miR-302c and low expression of miR-222 are associated with longer overall survival [214]. At the biological level, miR-302 cluster promoters are bound by key pluripotency transcription factors, including OCT4, SOX2, NANOG and TCF3, and these miRNAs are involved in the regulation of the G1–S transition and self-renewal of pluripotent stem cells [215]. In addition, the cluster miR-302/367 promotes BMP pathway activation by directly targeting multiple BMP inhibitors, such as *TOB2*, *DAZAP2*, and *SLAIN1*, thereby contributing to the maintenance of pluripotency and the regulation of differentiation programs [216]. miR-222 regulates *MMP1* expression and promotes cellular invasion through both cis- and trans-regulatory mechanisms [217].

Beyond its established role in EAC development, miR-375 has also emerged as a clinically relevant prognostic biomarker. An analysis of paired tumor and adjacent nontumor tissues from 100 EAC patients revealed that reduced levels of miR-375 in tumor tissue were strongly associated with a worse prognosis, independent of tumor stage, lymph node status, cohort type, and the administration of chemoradiotherapy (CRT) [218]. Among the experimentally validated targets of miR-375, MXI1, an antagonist of c-MYC [219], and JAK2 [220] are both involved in the regulation of cellular proliferation.

Multivariate analysis of global miRNA expression in primary tumors revealed different miRNAs, namely, miR-143, miR-145, miR-199a-3p, miR-199a-5p, miR-100, and miR-99a, whose overexpression was associated with reduced overall survival. Notably, miR-143 and miR-145 exhibited higher expression levels in tumors with poorer prognoses and in patients with lymph node metastases, suggesting that re-expression may be associated with a more aggressive phenotype [221]. miR-199a-5p suppresses EMT by targeting *SOX4* and inhibits NF-κB signaling, whereas both miR-199a-3p and miR-199a-5p regulate *FXR1* and *PXN*, affecting cell adhesion, proliferation, and motility [222]. Overall, miR-199a has emerged as a robust prognostic marker and has been previously associated with poor outcomes in other solid tumors [221,223].

miR-16-2 and miR-30e were identified as relevant from a prognostic perspective. High expression of miR-16-2 was associated with the presence of lymph node metastases and with reduced overall and disease-free survival [224]. At the molecular level, miR-16-2 is associated with the suppression of *RAR-β2*, which encodes a receptor involved in cellular differentiation, and with the antiproliferative effects of retinoic acid [225], supporting its role in promoting tumor progression. In parallel, miR-30e has emerged as a strong negative prognostic marker: its high expression correlates with reduced survival and an increased risk of recurrence [224]. More recently, miR-30e was shown to be transcriptionally induced by SOX2 and to act through the inhibition of *USP4*, resulting in reduced SMAD4 activity and increased expression and activity of CK2. Through this axis, miR-30e promotes proliferation, invasion, migration, and activation of EMT, ultimately contributing to a more aggressive tumor phenotype [226].

### 3.4. MicroRNAs as Biomarkers for Cancer Resistance in EAC

Research has demonstrated that miRNAs regulate chemoresistance in EAC. Hummel et al. reported that miR-148a upregulation consistently improved the response to cisplatin and 5-FU treatment in most chemotherapy-sensitive and resistant EAC cell lines. MSK1, PXR and de novo DNA methylation have emerged as potential mediators of this mechanism [161].

Similarly, miR-31 plays a role in modulating the cellular response to radiation. Alterations in the expression of miR-31, together with DNA repair genes regulated by this miRNA, were observed in tumors resistant to neoadjuvant CRT, suggesting that the miR-31-mediated regulation of DNA repair may constitute a major mechanism underlying therapeutic resistance [227].

Streppel et al. demonstrated that miR-223 was upregulated during progression from BE-ND to EAC in most patients and that increased miR-223 enhanced cisplatin sensitivity in OE-33 cells by reducing *PARP1* expression [228].

Low miR-187 expression is associated with resistance to CRT, increases the sensitivity of EAC to X-ray radiation and cisplatin, and is related to treatment failure in patients with EAC. miR-187 is significantly reduced in pretreatment-treated EAC tumors from patients who respond poorly to neoadjuvant CRT, suggesting that its downregulation may contribute to therapeutic resistance. Functional studies revealed that miR-187 overexpression modified the expression of 303 genes, including those involved in the complement cascade, and that C3 was increased in pretreatment biopsies of nonresponders, indicating that miR-187 is regulated in vivo. Overall, these results highlight the potential value of miR-187 and C3 as predictive markers of neoadjuvant CRT response in EAC patients [229].

In EAC patients who have a poor response to neoadjuvant CRT, the miR-17-5p level is reduced. Loss of miR-17-5p contributes to resistance to radiotherapy and neoadjuvant chemotherapy by derepressing target genes involved in survival and treatment response. In particular, miR-17-5p directly regulates *PRKACB* and *C6orf120*, whose increased expression has been associated with radioresistance and impaired treatment efficacy, potentially through the modulation of stress-response and immune-related pathways. In vitro, re-expression of miR-17-5p sensitized radioresistant OE33R cells to treatment [230].

Finally, the overexpression of miR-27b-3p increased the sensitivity of EAC cell lines to 5-FU and cisplatin. This effect has been associated with reduced expression of SP1 and PPARγ, a key pathway in chemoresistance and metastatic progression [231].

**Figure 2:**
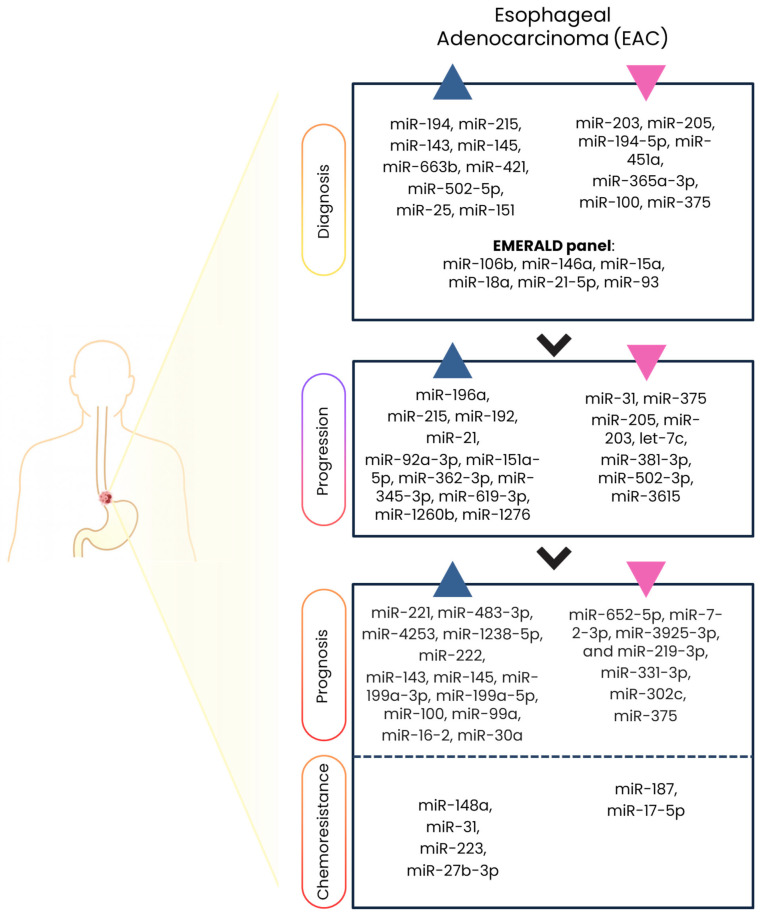
Summary of miRNA dysregulation in EAC. miRNAs whose expression is upregulated are indicated by blue arrowheads. miRNAs whose expression is downregulated are indicated by pink arrowheads; these miRNAs are associated with early detection and diagnosis, progression, prognosis, and resistance or sensitivity to chemoradiotherapy in EAC. (Created in BioRender. Cataldi-Stagetti, E. (2026) https://BioRender.com/o090zbo, accessed on 17 December 2025).

Table 2 and Figure 2 summarize the key miRNAs dysregulated in EAC across diagnostic, progression, prognostic, and therapy-resistance contexts.

## 4. MicroRNA-Based Therapeutic Approaches in Esophageal Cancer

Since miRNA expression is frequently deregulated in cancer, miRNAs represent promising therapeutic targets for novel molecular targeted therapies in tumor treatment [232].

Currently, miRNA-based therapies are based on two approaches: (i) inhibition of oncomiRs and (ii) restoration of downregulated tumor-suppressor miRNAs. This is achieved via two types of inhibitory molecules, anti-miRNA oligonucleotides (AMOs) and miR masks. AMOs act as competitive inhibitors, blocking the interaction between specific miRNAs and their target mRNAs [233]. Instead, miR masks are perfectly complementary to the miRNA binding site in a target gene and prevent the endogenous miRNA from accessing the binding site [234]. The re-expression of downregulated tumor-suppressor miRNAs can be accomplished via miRNA mimetics (short synthetic double-stranded RNA molecules) or miRNAs encoded by viral vectors and transduced into cells/tissues [232,235].

In EC, miRNA modulation has emerged as a clinically relevant strategy with potential applications in treatment prediction, therapeutic sensitization, and disease monitoring. Several miRNAs have been identified as key regulators of chemosensitivity, acting to sensitize cancer cells to chemotherapy. miR-29c sensitizes EC cells to 5-FU by regulating the FBXO31–p38 signaling pathway, and systemic administration of miR-29c oligonucleotides has been proposed as an innovative therapeutic approach to significantly reduce 5-FU chemoresistance [171].

Tumor suppressor miRNAs, such as miR-100, can suppress proliferation, migration, invasion, and tumor growth in vivo by targeting *CXCR7*, encoding a chemokine receptor implicated in tumor progression, angiogenesis, and metastasis [131].

Conversely, oncomiRs such as miR-455-3p drive chemoresistance and tumor recurrence; their silencing reduces T-IC subpopulations and simultaneously inactivates multiple stemness-associated pathways, including the Wnt/β-catenin and TGF-β/SMAD signaling pathways [167].

miRNAs have also increasingly emerged as targets of experimental anticancer drugs. Owing to their low toxicity to healthy cells and high selectivity for tumor cells, organometallic arene Ru(II) complexes are considered promising alternatives to cisplatin. Among these compounds, Rawq01 exerts antitumor activity in EC by downregulating miR-21, leading to the restoration of *PTEN* expression and the inhibition of the PI3K/AKT pathway [236].

Overall, these findings indicate that miRNAs involved in chemoresistance act not only as key molecular regulators but also as predictive biomarkers and therapeutic targets capable of simultaneously modulating multiple oncogenic pathways, supporting patient stratification and personalized therapeutic approaches [237].

The clinical translation of miRNA-based therapies is progressing, albeit with challenges. Several miRNA derivative clinical nucleotide drugs (mdCNDs), including MRX34 (miR-34a) [238], Miravirsen (miR-122) [239], and TargomiRs (miR-16) [240], have entered clinical trials, showing promising gene modulation and safety in patients with refractory tumors or viral infections. However, challenges such as enzymatic degradation, low cellular uptake, off-target effects, and immune reactions necessitate advanced delivery systems. Recent developments include lipid, polymeric, and metallic nanoparticles; self-assembling vectors; exosomes; and cell-penetrating peptides, which are often combined with stimulus-responsive release strategies (pH, redox, enzymes, ATP) [241]. Nanotechnologies have significantly improved miRNA delivery, using liposomes, dendrimers, polymeric nanoparticles, nanogels, and hybrid/exosome-mimetic systems to protect miRNAs from degradation and enable both passive and ligand-mediated targeting [242].

Currently, circulating miRNAs associated with chemosensitivity and resistance in EC suggest potential applications as noninvasive prognostic and predictive biomarkers, supporting early patient stratification and therapeutic monitoring [237,243].

## 5. Conclusions

Current evidence demonstrates that miRNAs are critically involved in the biology of EC. The integration of miRNA expression profiles with conventional clinicopathological parameters may contribute to improved early detection, more accurate risk stratification, and the development of personalized therapeutic strategies for both ESCC and EAC patients.

Several miRNAs, including miR-21, miR-375, miR-203 and miR-205, are consistently deregulated across both histological subtypes of EC, i.e., ESCC and EAC. Their recurrent alteration points to a role in shared, fundamental mechanisms of esophageal carcinogenesis, such as dysregulated proliferation, altered differentiation and enhanced invasive potential. The identification of these common miRNA signatures highlights their value as biomarkers capable of providing prognostic information regardless of histology and complementing existing staging systems while also supporting risk stratification irrespective of tumor histology.

Future large-scale, prospective and multicenter studies, together with the methodological standardization of miRNA detection and quantification, will be crucial to validate their clinical utility and enable their translation from experimental research into routine clinical practice.

## Figures and Tables

**Table 1:** Key microRNAs involved in ESCC diagnosis, progression, prognosis, and therapy resistance.

miRNA	Expression in ESCC	Sample	Main Targets/Pathways	Functional Role	Clinical Relevance	References
miR-10a, miR-22, miR-100, miR-148b, miR-223, miR-133a, miR-127-3p	All upregulated (miR-223 downregulated)	Serum	*SOX4*, *ARTN*, *VEGF-A*, *FGF2-FGFR2*, mTOR, Wnt/β-catenin, *CXCR7*, *PI3K/AKT*, *FBXW7*, *IGF1R*, *HIF-1α*	Angiogenesis, motility, EMT suppression, migration, proliferation	Diagnostic	[23,24,25,26,27,28,29,30]
miR-20b-5p, miR-28-3p, miR-192-5p, miR-296-5p	All upregulated	Serum	*PTEN*, *FBXW7* (PI3K/AKT pathway), *BIM*, *ARF6* (Hedgehog signaling)	Proliferation, migration, invasion	Diagnostic	[31,32,33,34,35,36,37,38,39,40,41]
miR-106a, miR-18a, miR-20b, miR-486-5p, and miR-584, miR-223-3p	All upregulated (miR-223-3p downregulated)	Serum	*PTEN* (PI3K/AKT pathway), *CDK4*, *BCAS2*	Proliferation, cell-cycle progression, apoptosis	Diagnostic	[32,42,51,54,55,103,107]
miR-8073, miR-3196, miR-744-5p, miR-6820-5p, miR-6794-5p, miR-6799-5p	miR-8073, miR-3196, miR-744-5p upregulated; miR-6820-5p, miR-6794-5p, miR-6799-5p downregulated	Serum	TGF-β signaling, PI3K/AKT, chromatin remodeling	Cell cycle regulation, angiogenesis, EMT, apoptosis, metastasis	Diagnostic	[43,44,45,46,47]
miR-21	Upregulated	Serum/tissue	*PTEN*, *PDCD4*, *TPM1*	Proliferation, invasion, resistance	Diagnostic, prognostic, predictive	[53,134,135,136,168,169,170]
miR-205-5p	Upregulated	Plasma	*PTEN*, *SMAD4*, PI3K/AKT pathway, TGF-β pathway	Proliferation, EMT	Diagnostic	[56,57,58,59,60]
miR-25	Upregulated	Serum	*TGF-β*, *VEGF*, *EGFR*, Wnt/β-catenin	Angiogenesis, metastasis	Diagnostic	[61,62]
miR-1322	Upregulated	Serum	*ECRG2*	Proliferation	Diagnostic	[63,64]
miR-16-5p, miR-451a, miR-574-5p	Upregulated	Serum	*RECK*, *SOX6*	Proliferation	Diagnostic	[50,65,66]
miR-375	Downregulated	Serum/tissue	*IGF1R*, *PDK1* (PI3K/AKT pathway), *XPR1* (BRAF-ERK1/2-p53), *PRDX1*	Proliferation, survival, metastasis	Diagnostic, progression, prognostic	[48,52,56,67,68,98,99,100,101,102,103,104,105,147,148,149]
miR-216a/b	Downregulated	Serum	*KRAS*, *eIF4B*, *ZEB1*, *IGF2BP2*	EMT, proliferation	Diagnostic	[70,71,72,73]
miR-613	Downregulated	Serum/tissue	*FN1*	Migration, invasion	Diagnostic	[74,75,76,77,78]
miR-1297	Downregulated	Serum	EphA2	Metastasis	Diagnostic	[79,80,81]
miR-29b	Downregulated	Tissue	*MMP2*	Invasion, metastasis	Progression	[85,86,87,88]
miR-30c	Downregulated	Serum/tissue	*SNAI1*, PI3K/AKT pathway	EMT suppression	Progression	[89,90,91,92,93]
miR-150	Downregulated	Tissue	*ZEB1*, *c-MYB*, *NOTCH3*, *CXCR4*	EMT/MET	Progression	[94,95,96,97]
miR-486-5p	Upregulated	Serum	*CDK4*, *BCAS2*	Cell-cycle progression, apoptosis	Progression	[34,106,107]
miR-630	Downregulated	Tissue	*SLUG*, *GAK*, *PRKCI*, JAK2/STAT3 pathway	EMT, invasion	Progression	[108,109,110,111,112,113]
miR-718	Downregulated	Plasma	*VEGF*, *HOXB8*	Proliferation, invasion, angiogenesis, apoptosis	Progression	[114,115]
miR-9	Upregulated	Plasma/tissue	α-catenin, β-catenin	EMT, metastasis	Progression	[118,119]
miR-155-5p	Upregulated	Tissue	*TP53INP1*	Proliferation	Progression	[120,121,122,123]
miR-183	Upregulated	Tissue	*PDCD4*, *SMAD4*	EMT, survival	Progression	[124,125,126]
miR-367	Upregulated	Plasma	*RAB23*	Proliferation	Progression	[127,128]
miR-100-5p, miR-203a-3p	Downregulated	Tissue	*CXCR7*, *FKBP5*, PI3K/AKT pathway	Invasion suppression	Progression	[129,130,131,132,133]
miR-133a	Downregulated	Tissue	*SOX4*, *IGF14*	Proliferation, migration, invasion	Prognostic	[137,138]
miR-133b	Downregulated	Tissue	JAK2/STAT3 pathway, *SNAIL1*	Survival, progression	Prognostic	[139,140,141]
miR-138	Downregulated	Serum/tissue	NF-κB	Proliferation, invasion	Prognostic	[142,143,144,145,146]
miR-1246	Upregulated	Serum	p53 network, *DYRK1A*	Stemness, invasion	Prognostic	[150]
miR-655	Downregulated	Tissue	*ZEB1*, *TGFBR2*	Migration, invasion	Prognostic	[151,152,153]
miR-203	Downregulated	Tissue	*TP63*	Survival, differentiation	Prognostic	[154]
miR-129	Upregulated	Tissue	*APC*, *RAB11*	Progression	Prognostic	[155]
miR-296	Upregulated	Tissue	cyclin D1, p27, *MDR1* (P-gp)	Proliferation	Prognostic	[156]
miR-142-3p	Upregulated	Tissue	*APC*, *KLF4*, *BCL2L1*	Proliferation	Prognostic	[157]
miR-27a	Downregulated	Cell lines	P-gp, *BCL2*	Drug resistance	Predictive	[160]
miR-148a	Upregulated	Cell lines	*DNMT1*, *DNMT3B*, *PXR*	Chemosensitivity	Predictive	[161]
miR-141	Upregulated	Cell lines	*YAP1*	Cisplatin resistance	Predictive	[162]
miR-23a	Upregulated	Plasma/tissue	*PTEN*, *CDH1*, *IRF1*, *APAF1*, *TOP2B*	Chemoresistance	Predictive	[163]
miR-218	Downregulated	Cell lines	Survivin	Chemosensitivity	Predictive	[164]
miR-455-3p	Upregulated	Tissue	Wnt/β-catenin pathway, TGF-β/SMAD pathway	Chemoresistance	Predictive	[165,166,167]
miR-29c	Downregulated	Serum/tissue	*FBXO31*	Chemoresistance	Predictive	[171]
miR-196b	Upregulated	Cell lines	*EPHA7/EPHA2*	EMT, resistance	Predictive	[172]
miR-192-5p	Upregulated	Plasma	*ERCC3*, *ERCC4*	Cisplatin resistance	Predictive	[173,174]

**Table 2:** Key microRNAs involved in EAC diagnosis, progression, prognosis, and therapy resistance.

miRNA	Expression in EAC	Sample	Main Targets/Pathways	Functional Role	Clinical Relevance	References
miR-194, miR-215, miR-143, miR-145	All upregulated	Serum/Tissue	*HNF1α*, *TP53*	Metaplasia acquisition	Diagnostic	[178,179,180,181,221]
miR-203, miR-205	Downregulated	Tissue	*TP63*	EMT	Diagnostic/progression	[180,190]
miR-375	Downregulated	Tissue, serum	*PDK1*, *IGF1R*, *MXI1*, *JAK2*	Proliferation	Diagnostic/progression/prognostic	[183,188,218,219,220]
miR-663b, miR-421, miR-502-5p	Upregulated	Tissue	*SMAD4*, *PTEN*, *c-MYC*, *BCL2*	Tumorigenesis	Diagnostic	[184]
miR-25, miR-151, miR-100, miR-375	miR-25, miR-151 upregulated; miR-100, miR-375 downregulated	Serum	*CDH1*, p21, Bim, *PDK1*, *IGF1R*, *MXI1*, *JAK2*	Proliferation, invasion	Diagnostic	[185]
miR-106b, miR-146a, miR-15a, miR-18a, miR-93, miR-21-5p	Dysregulated	Plasma	Cell-cycle regulation	Proliferation	Diagnostic	[186]
miR-196a	Upregulated	Tissue	*SPRR2C*, *S100A9*, *KRT5*, NF-κB, c-MYC	EMT, proliferation	Progression	[183,187,188]
miR-31	Downregulated	Tissue	*E2F2/CDKN2A*	Proliferation	Progression/predictive	[189,227]
miR-215, miR-192, miR-205, let-7c, miR-203	miR-215, miR-192 upregulated; miR-205, let-7c, miR-203 downregulated.	Tissue	*TP53*, *TP63*, *HMGA2*	Progression, EMT	Progression	[180,190]
miR-21	Upregulated	Tissue	*PDCD4*, *PTEN* (PI3K/AKT pathway)	Proliferation	Progression	[136,191]
miR-92a-3p	Upregulated	Serum/tissue	*PTEN*, *BIM*, TGF-β/SMAD pathway	Early progression	Progression	[192]
miR-221, miR-483-3p	Upregulated	Tissue	*MALAT1*	Invasion, recurrence	Prognostic	[194,195,196,197,198,199,200]
miR-652-5p, miR-7-2-3p	Downregulated	Tissue	SDC1/TGFβ2/pERBB4, *DCLK1*	EMT, proliferation	Prognostic	[201,202,203]
miR-331-3p	Downregulated	Serum	*HER2*, PI3K/AKT pathway	Proliferation, migration	Prognostic	[204,205,206,207,208]
miR-4253, miR-1238-5p	Dysregulated	Serum	*LHX2*	Proliferation, invasion, apoptosis	Prognostic	[209,210,211,212,213]
miR-222, miR-302c	Dysregulated	Serum	*TOB2*, *DAZAP2*, *SLAIN1*, *MMP1*	Invasion	Prognostic	[214,215,216,217]
miR-199a-3p, miR-199a-5p	Upregulated	Tissue	SOX4, FXR1, PXN	Proliferation	Prognostic	[221,222,223]
miR-16-2	Upregulated	Tissue	*RAR-β2*	Tumor progression	Prognostic	[224,225]
miR-30e	Upregulated	Tissue	*USP4*	EMT, invasion	Prognostic	[226]
miR-148a	Upregulated	Cell lines	*MSK1*, *PXR*	Chemosensitivity	Predictive	[161]
miR-223	Upregulated	Cell lines	*PARP1*	Chemosensitivity	Predictive	[228]
miR-187	Downregulated	Tissue	Complement cascade (C3)	CRT resistance	Predictive	[229]
miR-17-5p	Downregulated	Cell lines	*PRKACB C6orf120*	CRT resistance	Predictive	[230]
miR-27b-3p	Upregulated	Cell lines	*SP1*, *PPARγ*	Chemosensitivity	Predictive	[231]

## Data Availability

No new data were created or analyzed in this study. Data sharing is not applicable to this article.

## Abbreviations

The following abbreviations are used in this manuscript:

5-FU	5-Fluorouracil
AMOs	Anti-miRNA oligonucleotides
BE	Barrett’s Esophagus
BE-ND	Nondysplastic BE
CRT	Chemoradiotherapy
EC	Esophageal Cancer
EAC	Esophageal Adenocarcinoma
EMT	Epithelial–Mesenchymal Transition
ESCC	Esophageal Squamous Cell Carcinoma
GERD	Gastroesophageal reflux disease
HGD	High-grade dysplasia
LGD	Low-grade dysplasia
MET	Mesenchymal–Epithelial Transition
miRNA	MicroRNA
oncomiRs	Oncogenic microRNAs
T-ICs	Tumor-initiating Cells
